# Severe Angle Class III skeletal malocclusion associated to mandibular prognathism: orthodontic-surgical treatment

**DOI:** 10.1590/2177-6709.21.6.103-114.bbo

**Published:** 2016

**Authors:** Marcelo Quiroga Souki

**Affiliations:** 1 Certificate and MSD in Orthodontics, PUC-Minas. Brazilian Board of Orthodontic Diplomate.

**Keywords:** Angle Class III malocclusion, Orthodontic treatment, Orthognatic surgery

## Abstract

The present case report describes the orthodontic treatment of a young adult patient (18y / 1m), Class III skeletal malocclusion, with mandibular prognathism and significant dental compensation. The canine relation was Class III, incisors with tendency to crossbite and open bite, moderate inferior crowding, and concave profile. Skeletal correction of malocclusion, facial profile harmony with satisfactory labial relationship, correction of tooth compensation and normal occlusal relationship were obtained with orthodontic treatment associated to orthognathic surgery. This case was presented to the Brazilian Board of Orthodontics and Facial Orthopedics (BBO), as part of the requirements to become a BBO diplomate.

## RESUMO

O presente caso clínico descreve o tratamento ortodôntico de um paciente com 18 anos e 1 mês de idade, portador de má oclusão esquelética de Classe III, com prognatismo mandibular e significativa compensação dentária. A relação entre caninos era de Classe III, incisivos com tendência à mordida cruzada e mordida aberta, moderado apinhamento inferior, além de perfil côncavo. A correção esquelética da má oclusão; a harmonia do perfil facial, com relação labial satisfatória; correção da compensação dentária e relação oclusal normal foram obtidas com o tratamento ortodôntico associado à cirurgia ortognática. Esse caso foi apresentado à Diretoria do Board Brasileiro de Ortodontia e Ortopedia Facial (BBO), como parte dos requisitos para a obtenção do título de Diplomado pelo BBO.

## INTRODUCTION

An 18-year-old patient, male, sought orthodontic treatment to correct the occlusion. His main complaint was related to his chin size and the lower teeth positioned in front of the upper incisors. During the anamnesis, he reported having had similar cases among his relatives. No systemic and/or medical abnormalities were described. His oral hygiene was unsatisfactory, however, with no previous history of caries and significant periodontal disease. 

## DIAGNOSIS

**Figure 1:**
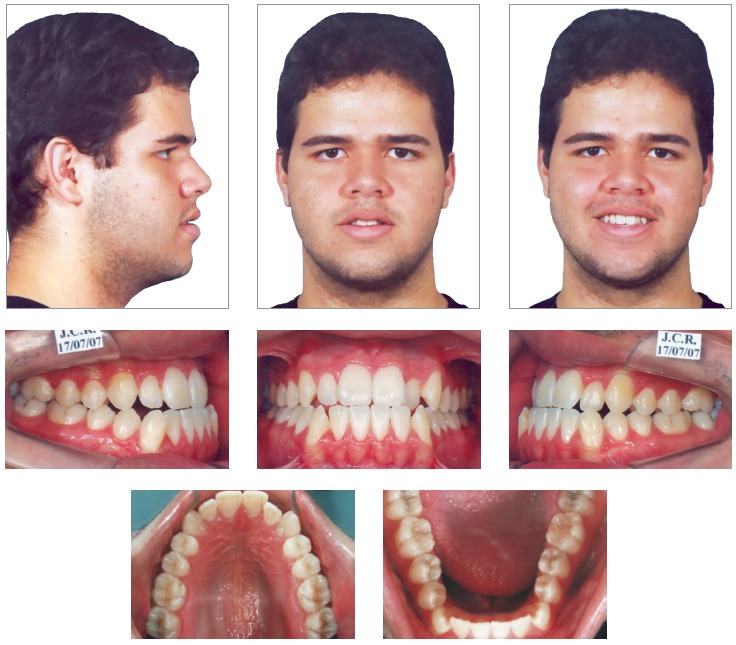
Initial facial and intraoral photographs.

The clinical examination showed, in frontal view, a symmetrical face and a reduced exposure of the upper incisors upon smiling. In the lateral view, the profile was concave, associated to an incompetent lip seal at rest. The upper lip was well positioned (Upper lip - S-line = 0.5 mm), while the lower lip was protruded (Lower Lip - S-line = 4 mm) (Fig 1).

**Figure 2:**
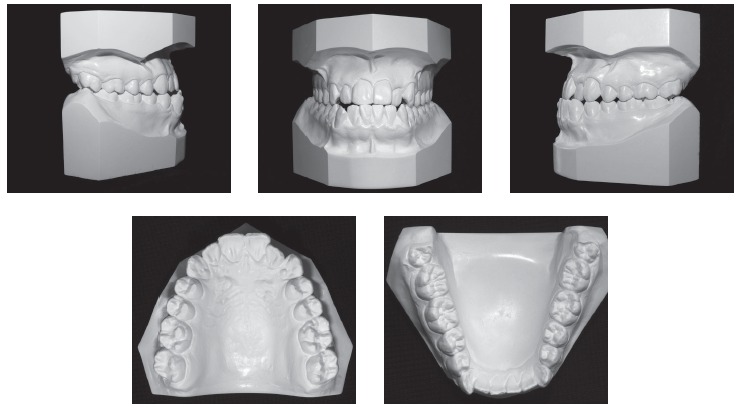
Initial dental casts.

During the intraoral clinical examination, bilateral Angle Class III malocclusion was observed, associated with significant dental compensation and moderate crowding in both arches. The upper incisors were proclined (1.NA = 30^o^ and 1-NA = 11mm), while the lower incisors were vertical (1.NB = 22^o^ and IMPA = 83 ^o^). Overbite and overjet were reduced, with tendency to open bite and crossbite in the anterior segment (Figs 1 and 2).

**Figure 3:**
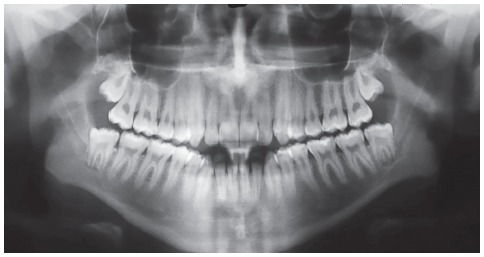
Initial panoramic radiograph.

**Figure 4:**
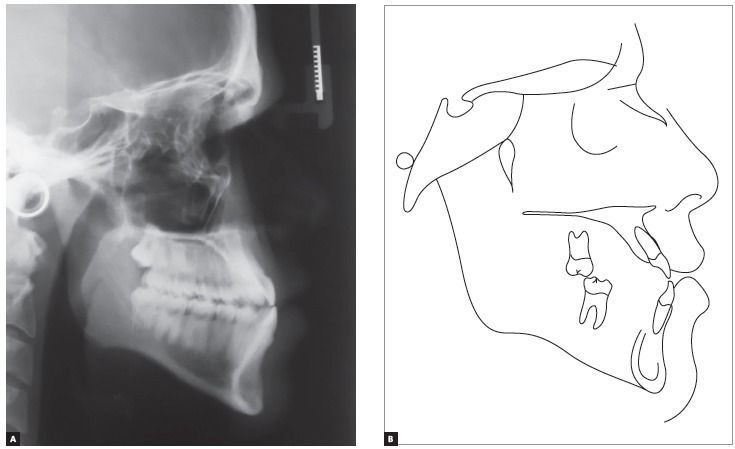
Initial cephalogram (A) and cephalometric tracing (B).

Panoramic radiograph indicated the presence of all permanent teeth, including the third molars. Few dental elements presented with restorations, but all with satisfactory aspect. Supporting bone structures presented adequate levels (Fig 3). The cephalometric evaluation indicated Class III skeletal malocclusion (ANB = -3 °), associated with mandibular prognathism (SNB = 87 °) and maxilla positioned within normal standards (ANS = 84 °). In the vertical aspect, the patient presented measures indicative of normal growth pattern (SN.GoGn = 30 ° and FMA = 23 °) (Fig 4).

## TREATMENT PLAN

With the aim of correcting skeletal malocclusion, achieve harmony of the facial profile, allow satisfactory labial relationship and passive lip seal, correct the dental compensation created by malocclusion and achieve a normal occlusal relationship, an orthodontic treatment associated with the surgical correction of skeletal malocclusion through orthognathic surgery was proposed. Initially, the patient was referred to the buco-maxillofacial surgeon for surgical planning, preliminary orientations and decision about the upper and lower third molars. After his return, the fixed upper and lower appliances were installed, with Roth prescription brackets (0.022” x 0.028”), first molar bands with double convertible tube and second molars with single tubes in both arches. For dental leveling and alignment, the following orthodontic archwire sequence was planned: 0.014” NiTi wire; 0.016”, 0.018” and 0.017” x 0.025” stainless steel archwire with ideal torques. After leveling and alignment conclusion and correction of dental inclinations, dental impressions were taken to help the presurgical preparation with the necessary wire bends. A surgical 0.019” x 0.025” archwire with welded hooks were programmed. After the orthognathic surgery, the need for the use of intermaxillary elastics and wire folds for the conclusion of the case was evaluated.

## TREATMENT PROGRESS

**Figure 5:**
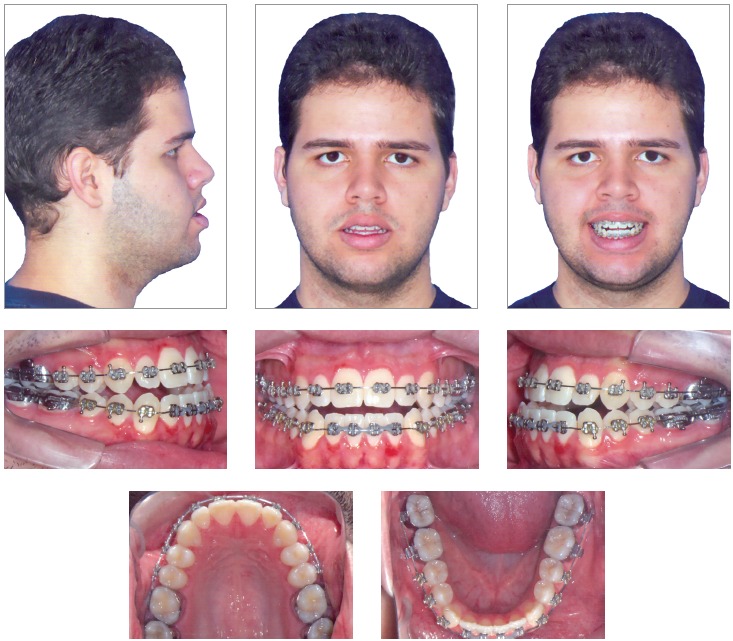
Intermediate facial and intraoral photographs: presurgical phase.

**Figure 6:**
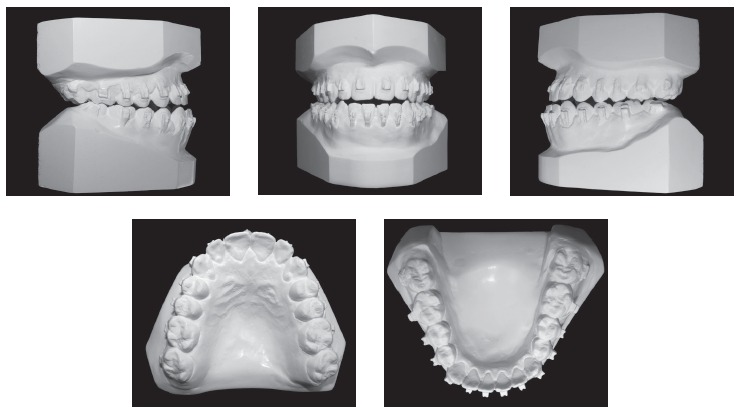
Intermediate: presurgical phase dental casts.

**Figure 7:**
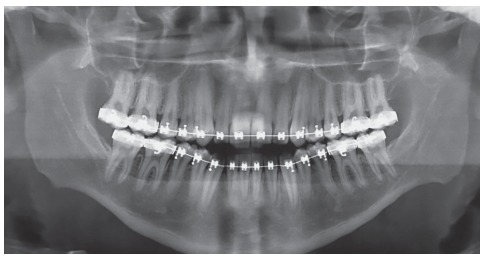
Intermediate: presurgical phase panoramic radiograph.

**Figure 8:**
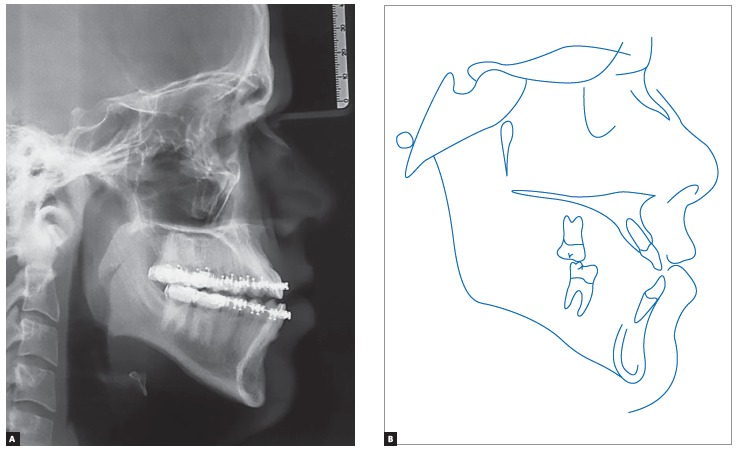
Intermediate: presurgical phase cephalogram (A) and cephalometric tracing (B).

The proposed treatment plan was followed as planned until the end of leveling and alignment. At this stage, it was perceived a great difficulty to obtain the ideal transverse arch positioning due to the occlusal interference created by the significant compensation presented by the patient original occlusion. Thus, an acetate plate was adapted in the upper arch, with occlusal bite-block, to eliminate the occlusal locking and facilitate the posterior segment torque movement in the lower arch, with greater efficiency. Subsequently, dental casts were performed and the final preparation of the preoperative surgical preparation with the necessary bends was performed. After the pre-surgical orthodontics conclusion (Fig. 5 to 8), the final surgical procedure plan was defined, along with the maxillofacial surgeon. The surgical arches 0.019”X0.025” were installed with welded hooks, tied with steel wires in all the teeth. To achieve the best possible facial and skeletal results, Le Fort I maxillary surgery, with posterior impaction and clockwise rotation was performed, combined with sagittal mandibular osteotomy and an anterior and vertical mentoplasty.

## TREATMENT RESULTS

**Figure 9:**
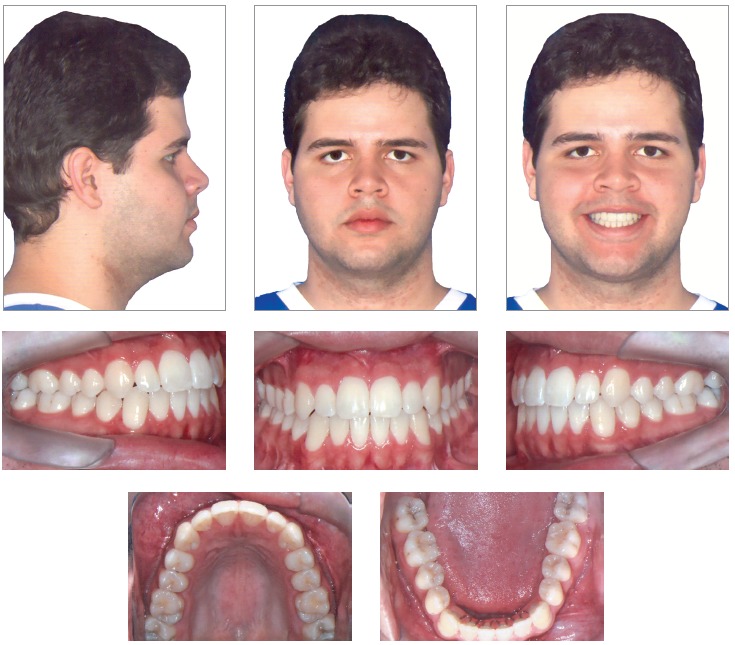
Final facial and intraoral photographs.

A significant improvement was seen in facial esthetics. A pleasant facial smile was obtained, with an increase in the upper teeth exposure. The facial profile became straight, with improvement in the lip contour, as well as obtaining an appropriate lip seal due to reduction of the protrusion of the lower lip (reduced from 4 mm to 0 mm, to the Steiner’s S line) (Fig. 9 and Tab 1).

**Table 1:**
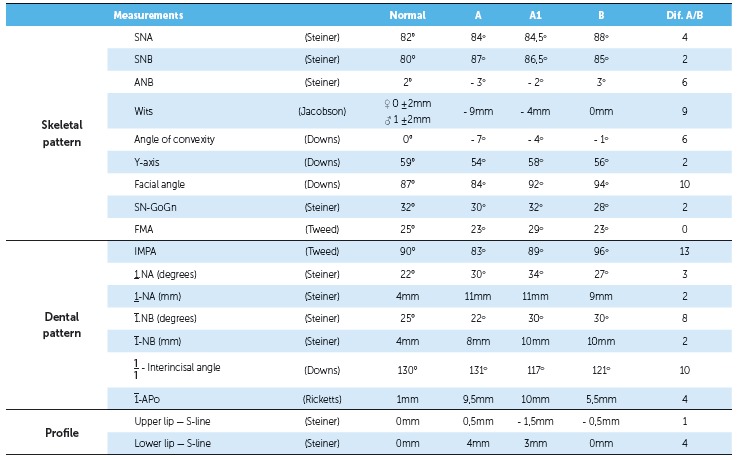
Cephalometric measurements: initial (A), pre-surgery (A1) and final (B).

**Figure 10:**
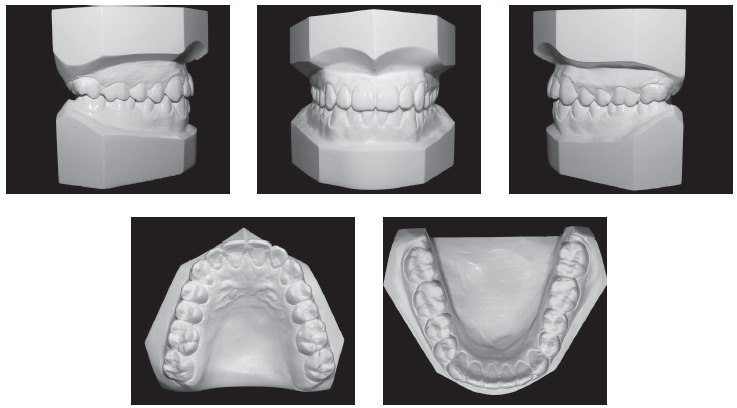
Final dental casts.

Intraoral records (Figs 9 and 10) demonstrated a satisfactory occlusal relationship in a lateral view, with good intercuspation and solid molar and canine Class I relationship. From the frontal view, it was possible to observe the dental midline coincidence and an adequate overbite. Because of the improvement in occlusion, the patient presented anatomical conditions to obtain satisfactory occlusal guides, with lateral movement guided by the canines and protrusive by the incisors.

**Figure 11:**
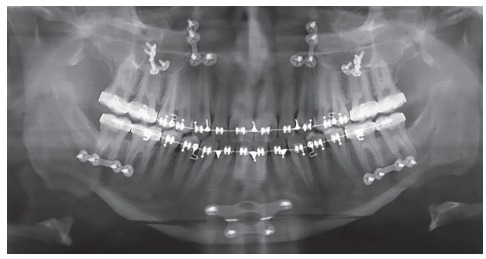
Final panoramic radiograph.

**Figure 12:**
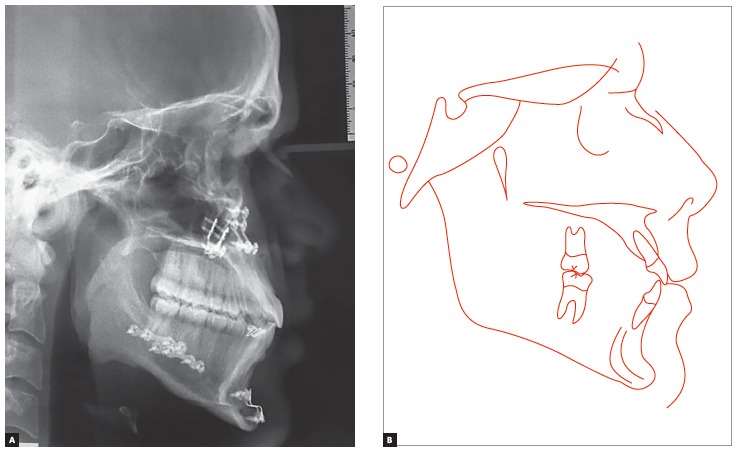
Final cephalogram (A) and cephalometric tracing (B).

**Figure 13:**
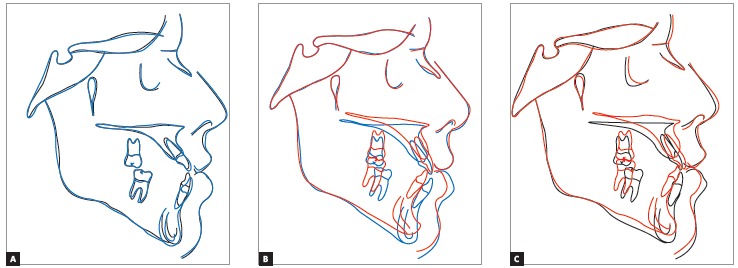
Total superimpositions of cephalometric tracings: (A) initial (black) and pre-surgery (blue); (B) pre-surgery (blue) and final (red); (C) initial (black) and final (red).

**Figure 14:**
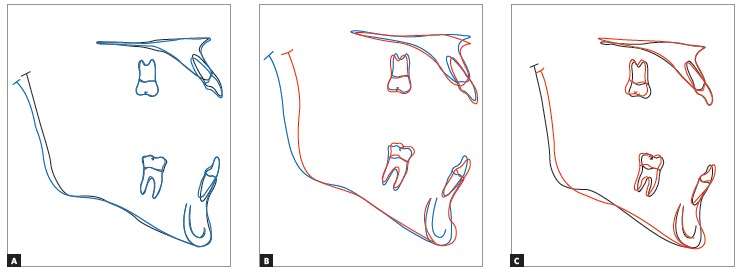
Partial superimpositions of cephalometric tracings: (A) initial (black) and pre-surgery (blue); (B) pre-surgery (blue) and final (red); (C) initial (black) and final (red).

Cephalometric superimpositions showed a significant improvement in the maxillary position, due to an advance and impaction of the posterior segment of the maxilla. A mandibular posterior displacement and an anterior and vertical repositioning of the chin were observed in the mandible. These modifications allowed an improvement in sagittal skeletal disharmony, with a reduction of 6 degrees in the ANB angle (Steiner) from -3^o^ to 3^o^ and in Wits (Jacobson) measurement from -9 mm to 0 mm (Table 1, Figs 11 to 14).

## FINAL CONSIDERATIONS

The Angle Class III malocclusions can present a variable severity, with different levels of resolution complexity. In general, the greater the skeletal involvement, the more complex the orthodontic treatment becomes^1^. When the patient presents skeletal and dental disharmonies, usually, a significant facial impairment, with a consequent psychosocial impact is expected^2^ ^,^ ^3^. During the growth period, it is possible to establish orthopedic therapy to harmonize maxillomandibular growth^4^ ^,^ ^5^. However, when the approach is too late, in post-pubertal stage, with no growth potential, the options for treating these skeletal malocclusions become limited. Basically, dental compensation can be planned without skeletal disharmony correction or a complete orthodontic therapy correction associated with orthognathic surgery^6^ ^-^ ^9^. In the case described, the patient and his relatives complained about, not only the dental aspect, but also the facial disharmony. For this reason, an ortho-surgical approach was proposed to correct skeletal malocclusion and to harmonize the face. After the treatment plan presentation and explanation by the surgeon about the questions related to the surgical procedure, orthodontic treatment was started. The preoperative phase was adequate, highlighting only the difficulty in the transverse arch coordination due to a bite interference during the torque movements in the lower arch. For that reason, it was necessary to use a removal bite-block in the upper arch to release lower tooth movement. The result achieved by the surgery was very favorable, making easy the post-surgical phase and orthodontic ending. In sequence, the patient was referred to speech pathology work to adapt the muscular structures and the oral functions. The final treatment results were highly positive, reaching all the proposed objectives: significant improvement in facial profile and esthetics, in addition to functional and harmonic occlusion.
